# “Will It Affect Our Chances of Having Children?” and Feeling “Like a Ticking Bomb” —The Fertility Concerns and Fears of Cancer Progression and Recurrence in Cancer Treatment Decision-Making Among Young Women Diagnosed With Gynaecological or Breast Cancer

**DOI:** 10.3389/fpsyg.2021.632162

**Published:** 2021-06-02

**Authors:** Aleksandra Sobota, Gozde Ozakinci

**Affiliations:** University of St Andrews, School of Medicine, St Andrews, United Kingdom

**Keywords:** fear of cancer recurrence, fear of cancer progression, fertility, treatment decision-making, common-sense model, breast cancer, gynaecological cancer, shared decision-making model

## Abstract

**Objective:** Cancer treatment decision making process is particularly fraught with challenges for young women because the treatment can affect their reproductive potential. Among many factors affecting the process, fears of cancer progression and recurrence can also be important psychological factors. Our aim is to apply Common-Sense Model and shared decision-making model to explore experiences of treatment decision-making women of reproductive age who were diagnosed with gynaecological or breast cancer and the influence of fertility issues and fears of cancer progression and recurrence.

**Method:** We conducted telephone interviews with 24 women who were diagnosed with gynaecological or breast cancer aged 18–45, who finished active treatment within 5 years prior to study enrolment and had no known evidence of cancer recurrence at the time of participation. They were recruited from three NHS oncology clinics in Scotland and online outlets of cancer charities and support organisations. We analysed the data using Braun and Clarke's thematic analysis method as it allows for both inductive and deductive analyses.

**Results:** We identified five main themes pertaining to treatment-related decision-making experiences and fertility issues and fear of progression and recurrence: *Becoming aware of infertility as a potential consequence of cancer treatment; Balancing-prioritising cancer and fertility; Decisions about treatments; Evaluation of treatment decisions;* and *The consequences of treatments*. Sub-themes have also been reported. Different factors such as whether the cancer is breast or gynaecological, physicians' willingness of discussing fertility, influence of others in decision-making, childbearing and relationship status as well as fear of cancer recurrence emerged as important.

**Conclusion:** The importance of physicians directly addressing fertility preservation in the process of treatment decision-making and not treating it as an “add-on” was evident. Satisfaction with treatment decisions depended on both the quality of the process of decision making and its outcome. Fear of recurrence was present in different parts of the adaptation process from illness perceptions to post-treatment evaluation of decisions. Both Common-Sense Model and shared decision-making model were helpful in understanding and explaining young women's experience of treatment decision-making and fertility concerns.

## Introduction

The incidence of cancer in the United Kingdom has been increasing since the early 1990s (Cancer Research UK, 2020a). Notably among adults aged 25–49, cancer rates between 1993 and 2017 have increased by 21% (Cancer Research UK, 2020a). In this cohort, women were significantly more likely than men to be diagnosed with cancer, and between 2015 and 2017, four out of 10 were diagnosed with breast cancer (Cancer Research UK, 2020a). Cervical, ovarian, and endometrial cancer accounted for 8, 5.2, and 2.6% of all cancers in this group, respectively (Cancer Research UK, 2020a,b,c). Overall, 60% of women aged 25–49 diagnosed with cancer in the UK between 2015 and 2017 faced a disease that could have a considerable impact on their reproductive potential, either because of the disease itself or because of potential consequences of treatment.

The impact of cancer treatments on fertility contributes to poorer psychological well-being including higher levels of distress (Sobota and Ozakinci, 2018; Logan et al., 2019) and decreased quality of life (Sobota and Ozakinci, 2014) among young women with cancer. Oncofertility has emerged as a novel field to address fertility needs of young people with cancer. Research has shown the beneficial effect of discussing fertility preservation as part of oncology consultation (Ussher et al., 2018) and providing young women with decision aids to facilitate decision making around fertility preservation (Wang et al., 2019). While fertility preservation is increasingly available to cancer patients, with national guidelines acknowledging its importance and encouraging to provide the service (National Institute for Health Care Excellence, 2017; Ethics Committee of the American Society for Reproductive Medicine, 2018; Oktay et al., 2018), multiple barriers to accessing fertility preservation including age, relationship status, timing of decisions, and institutional factors still exist (Crawshaw et al., 2009; Lee et al., 2011; Niemasik et al., 2012; Yee et al., 2012; Hershberger et al., 2013a,b; Kirkman et al., 2013; Ruddy et al., 2013; Snyder and Tate, 2013; Corney and Swinglehurst, 2014; Corney et al., 2014; Garvelink et al., 2015).

Another important factor contributing to decisions about fertility preservation is fear of cancer progression and recurrence (Gorman et al., 2011; Lee et al., 2011; Hershberger et al., 2013b; Kirkman et al., 2013; Snyder and Tate, 2013; Garvelink et al., 2015). Fear of cancer progression and recurrence is the “fear, worry or concern relating to the possibility that cancer will come back or progress” (Lebel et al., 2016; p. 3,265). It can affect patients' treatment choices and in a study among breast cancer patients, Stafford et al. (1998) have demonstrated that fear of cancer recurrence was one of the main reasons women tended to choose more radical treatment.

Considering the complexity of cancer treatment-related decision making, we conducted a qualitative study exploring the experiences of treatment decision-making of young women diagnosed with breast or gynaecological cancer and the influence of fertility issues and fears of cancer recurrence and progression on these decisions.

We chose to apply two theoretical models—the Common-Sense Model (CSM) (Leventhal et al., 2004) and shared decision-making model (Elwyn et al., 2012) to study the decision-making processes. In this article, we are reporting on our findings relating to the importance of fertility, fear that cancer may recur or progress, and illness perceptions as defined by CSM on treatment-related decision making, however the broader project has findings that relate to other aspects of the CSM and decision-making process.

The CSM is a model of self-regulation widely used to study illness perceptions and management. The model asserts that in response to a health threat, the individual forms both cognitive representations (illness perceptions) and emotional representations (e.g., fear and worry) of the threat. There are five dimensions to illness perceptions: identity (the label for the threat and the symptoms), perceived cause, perceived timeline, perceived consequences, and perceived curability/controllability of the threat. Both of the representations mean that the individual goes through a process of developing coping procedures which are then evaluated in an appraisal process of whether they worked or not. It has been shown that patients' treatment decision-making processes are frequently driven by their own lay perceptions of illness and treatment (Charles et al., 1998).

Shared decision-making model has also relevance in the context of young women's experience of treatment decision-making and consideration to be given to fertility preservation. The involvement in the process of treatment decision-making can be an empowering experience for the patient (Whitney et al., 2004) and another strategy to cope with cancer. What is crucial in the shared decision-making model is the bidirectional exchange of information whereby the physician shares his or her medical knowledge as well as the opinions about different treatment modalities with the patient and the patient in turn provides the information about his or her values and preferences regarding treatments as well as sharing the pre-existing knowledge he or she has about his or her condition. Once the information exchange process has taken place, both the patient and the physician enter the deliberation stage where information is discussed in an interactional manner. In this process of negotiation, a decision regarding treatment is reached and implemented (Charles et al., 1999).

## Materials and Methods

### Design

Eligible women were invited to participate in an interview and were approached at the time of their outpatient clinic appointment in three UK-based hospitals (Edinburgh, Dundee, Kirkcaldy) or via online outlets of UK-based cancer charities (between October 2014 and May 2015). Based on Cancer Research UK and Office of National Statistics data (Local Government Association, 2015; Cancer Research UK, 2020a,b,c), the cumulative incidence of breast, ovarian, cervical, and womb cancer was ~97 in 100,000 women aged 25–49 in 2015. With numbers of potentially eligible women being small we anticipated we may encounter difficulties recruiting for the study, therefore we used this mixed approach strategy (NHS and cancer support organisations) to (1) reach a wider patient population and maximise recruitment potential, and (2) attempt to recruit as diverse and representative a sample of women as possible.

Women who consented to take part were interviewed over the phone. Braun and Clarke thematic analysis 2006 was used to explore women's experiences of cancer treatment-related decision-making.

This study has been reported in accordance with the COREQ criteria (Tong et al., 2007) (see Appendix 1). See the interview guide in Appendix 2.

### Participants

Women meeting the following inclusion criteria were invited to participate:

- ∙ received a diagnosis of breast or gynaecological cancer between the ages of 18–45 years old;
- ∙ were menstruating at the time of diagnosis;
- ∙ had chemotherapy (neo-adjuvant or adjuvant) as part of their treatment if they were diagnosed with breast cancer;
- ∙ finished active treatment (with the exception of endocrine therapy for breast cancer) within 5 years prior to study enrolment;
- ∙ had no known evidence of cancer recurrence at the time of participation;
- ∙ spoke English or Polish.

We chose to focus on breast and gynaecological cancers for several reasons. First, these are some of the most common cancer diagnoses among women aged 25 to 49 (Cancer Research UK, 2020a,b,c). Second, their treatments can either impair fertility, or make one's fertility status post-treatment uncertain. As the literature seems to suggest that it is women's subjective perception of, rather than the objective fertility status that affect's women's well-being (Sobota and Ozakinci, 2014), we decided to include women who had any treatment with the potential to affect fertility. This included women who had chemotherapy for breast cancer as it is associated with uncertainty regarding individual fertility which may be difficult to predict (Wallace et al., 2005; Knobf, 2006; Duffy and Allen, 2009); women with early stage cervical cancer who underwent a cone biopsy or trachelectomy because while both treatments preserve fertility, they can be associated with adverse obstetric outcomes such as second trimester miscarriage (with a rate nearly twice as high as for the general population), and pre-term birth (Tirlapur et al., 2017). Also, according to the recent review, up to 61% of women who had a trachelectomy need artificial reproductive technologies to conceive (Tirlapur et al., 2017) which can be associated with uncertainty about one's fertility. Both atypical hyperplasia and early stage endometrial cancer (often grouped together), can be treated with progestogens, however, fertility outcomes are poorer than in the general population (Wei et al., 2017). The gold standard treatment for endometrial cancer—hysterectomy and bilateral salpingo-oophorectomy (Amant et al., 2018), will have the same impact on women's fertility as cytoreductive surgery for ovarian cancer, which is by far the most common surgical treatment for ovarian cancer due to the delay in its diagnosis (Cancer Research UK, 2017).

Although childbearing status affects the degree of importance women attach to fertility when diagnosed with cancer (Gorman et al., 2010; Canada and Schover, 2012; Ruddy et al., 2014) we did not include it in our inclusion and exclusion criteria and decided to recruit both women who did and those who did not have children prior to diagnosis. This was to achieve a wide variety of experiences and data saturation.

Participants were provided with a standard research pack including a cover letter, a participant information sheet, an opt-in form (not for participants recruited online), two copies of the consent form, an interview schedule, a debriefing form and two stamped-addressed envelopes. Women who opted in to take part were then contacted via phone to fill out the consent form and agree the interview date.

To recruit for the study, we relied on convenience sampling. This was to decrease the pressure to participate in the project investigating sensitive topics. Nonetheless, our sampling strategy yielded participants with a wide range of characteristics in terms of cancer diagnoses and treatments, age, relationship and childbearing status, and the use of fertility preservation. While the inclusion criteria indicate that Polish-speaking women would be included in the study, these participants were not actively sought. Although two were approached for participation, they decided not to participate.

Overall, 56 women expressed interest in taking part in the study and 24 were recruited (10 via clinics and 14 via online outlets; participation rate = 43%). Thirty-two women who initially expressed interest in participating, but did not make further contact with the research team were not re-approached, hence the reasons for non-participation remain unknown.

### Data Collection

All women were interviewed by the same researcher (AS) by phone. Interview by phone was selected primarily to facilitate data collection from participants who were recruited via online outlets and thus could potentially live in any part of the UK but they also possess other merits important for this study. This mode of interviewing allows the participant to remain anonymous, permits privacy, diminishes social pressure, and thus enables participants to disclose sensitive or intimate information more freely (Novick, 2008).

The interviews were guided by an interview schedule (see Appendix 2). Each interview started with an opening question asking the participant to describe the circumstances of her cancer diagnosis and the treatment process. Each interview ended with an open-ended question and this is where participants had a chance to speak about other issues they faced because of cancer diagnosis at a young age. Answers yielded additional themes that were not directly related to the research questions yet enriched the understanding of the participants' cancer experiences. Participants were asked to provide basic socio-demographic details (current age, country of origin, relationship status, childbearing status, monthly income before tax, and the highest education level) and disease characteristics (type of cancer, stage of cancer at diagnosis, types of cancer treatment received and date of diagnosis) if these were not mentioned during the interview. The interviews lasted on average 55 min (range = 22–121 min).

All interviews were digitally recorded and transcribed verbatim. Identifying details were removed from the transcripts and each transcript was assigned a numeric code. Notes taken during the interviews and reflections written after the interviews were assigned the same numeric code to link all the relevant participant data.

### Analysis

The data were analysed using the principles of thematic analysis outlined by Braun and Clarke (2006) which was conceived as a standalone data analysis method for “identifying, analysing, and reporting patterns (themes) within data” (p. 79). The analysis followed six steps of: (1) Familiarisation with data, (2) Generation of initial codes, (3) Searching for themes, (4) Reviewing themes, (5) Defining and naming themes, and (6) Producing the report.

One of the important advantages of thematic analysis is its flexibility. As opposed to other methods of qualitative analysis it is not tied to any particular epistemological or theoretical approach (Braun and Clarke, 2006). It can, therefore, be adapted to the researcher's needs in terms of epistemological or theoretical framework. Another benefit of thematic analysis lies in the fact that, while many qualitative methods are purely inductive, thematic analysis can be used in both an inductive and deductive manner. A method that would allow for a deductive approach and application of specific theoretical frameworks to the data was essential for this study and thematic analysis fulfilled these criteria. The CSM was chosen from the outset and guided both the design and the analysis of the data while shared decision-making model was selected to guide the data analysis.

Notably, Braun and Clarke's thematic analysis can be used as a realist method focusing on peoples' personal experiences and the meanings they attach to their lived realities, a constructionist approach where these meanings and experiences are considered an effect of discourses operating in society, or finally a contextualist approach which sits between realism and constructionism, and acknowledges that meanings and experiences, while grounded in one's reality are also a factor of broader societal and cultural constructs (Braun and Clarke, 2006).

In the inductive stage of our data analysis, we assumed a realist approach. As a paradigm it is often used to “discover knowledge” and purports that peoples' accounts reflect reality (Madill et al., 2000). By adopting it we aimed to tap into our participants' realities and “discover [categories/codes] within the data” (Madill et al., 2000). Therefore, the derived codes reflected our participants' understanding of their experiences and decision-making processes during cancer treatment in the wider context of preserving fertility and fear of cancer recurrence. At the end of this step, these initial codes were categorised into patterns, and patterns were then used as a foundation to which we applied our theoretical lens.

In the deductive stage of the analysis, we applied theoretical frameworks rooted in cognitive psychology of health and illness (the CSM) and health-related decision making (the shared decision-making model) to the patterned codes whereby moving our analysis into the contextualist territory. Contextualism postulates that all human experience is context specific and subjective, and that phenomena can be interpreted in multiple ways (King and Brooks, 2016). These interpretations depend on the specific context of research and the stance of the researcher. As such, based on the aforementioned theories, we derived the final themes from the codes. This approach positions our analysis within contextual thematic analysis spectrum. This type of analysis has been successfully used within (Fielden et al., 2011; Faric et al., 2019) as well as outside of health research (Goldingay et al., 2018).

From the practical perspective, the interview transcripts were first read and reread for a thorough familiarisation with the data. Next, all the transcripts were uploaded to QSR International's NVivo 10 Software (NVivo qualitative data analysis Software, 2012) and the first cycle coding method—the descriptive coding—was applied to the data. Descriptive coding uses short phrases to summarise topics reoccurring in the data (Saldaña, 2015). Once all the data were coded, the second cycle coding method—the pattern coding—was applied. The pattern coding allows for grouping of the descriptive codes and making sense of the relationships among them (Saldaña, 2015). Through further reading and rereading of the interviews, secondary codes were refined to better reflect the data. Up to this point the data analysis was conducted by one researcher (AS). In the next step, the map of secondary codes was applied to three out of 24 interviews by the second researcher (GO). Where discrepancies in coding between the researchers occurred, these were discussed until a consensus was reached and codes were clarified and reorganised to better fit the data.

The analysis up to this point was carried out in an inductive manner. However, since this study focused particularly on the experiences of treatment-related decision-making and was driven by two theoretical models, once the secondary codes were obtained, the rest of the analysis was conducted in deductive manner.

This type of analysis, as suggested by Braun and Clarke (2006), focuses on answering a particular research question and exploring the theory, rather than on providing the description of the whole dataset (understood as all the data collected for the particular project). This approach results in a more detailed analysis of certain aspect of the dataset—in this case the data related to treatment decision-making in the context of fears of cancer progression and recurrence, and fertility. Therefore, at this point in analysis, all the codes were reviewed again and those that did not contribute directly to answering any of the research questions were moved to a separate folder. The remaining codes were iteratively reread and arranged according to the main concepts involved in the CSM and shared decision-making model. Codes categorised as belonging to the same concepts within the theoretical models were then conceptualised into internally homogenous and externally heterogeneous themes. These themes were subsequently discussed within the research team to assure the credibility and the rigour of the analysis.

**Figure 1:**
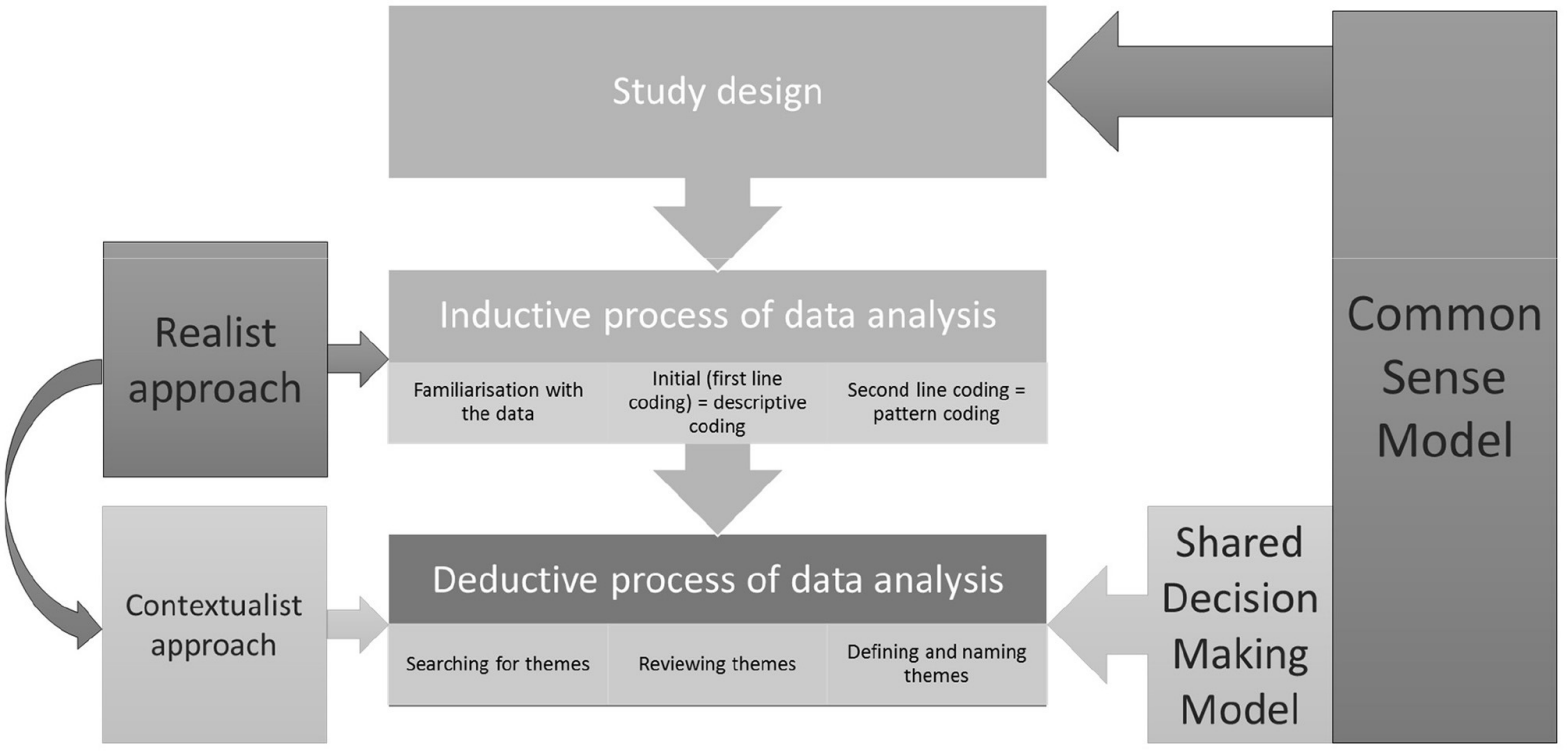
Data analysis process.

In summary, we initially approached data analysis from a realist perspective—looking for participants literal experience of cancer-treatment decision making, and moved into the contextualist territory—applying the theoretical lens of CSM and shared decision making model to contextualise the data. At the same time, our analysis progressed from an inductive to a deductive one. The CSM informed the study design (interview schedule), and both inductive and to a greater extent deductive data analysis, while the shared decision-making model was applied to the deductive part of the data analysis process. These processes are represented in Figure 1.

### Reflexivity Statement

Qualitative inquiry involves a degree of subjectivity and a neutral observer as such does not exist (Malterud, 2001), it is therefore important to outline the researcher's own effect on the process of data collection and analysis.

The data for this study was collected and primarily analysed by AS. This constituted part of her PhD project and all participants were made aware of this. AS approached this study bringing in both a personal and a professional perspective, the latter including that of a junior doctor, psychologist, and researcher. They have all contributed to how this project was conducted and are discussed below.

Personally, AS acknowledges that her attitude toward motherhood is rather ambivalent and she is unsure whether she wishes to ever have children. However, she believes that motherhood may be an important experience and one that, if missed, may cause the feeling of regret. With this in mind, AS found herself emotionally affected by participants' storeys. With some women being very close in age to AS, it proved difficult for her to completely distance herself from the extremely complex decisions these women had to make. Perhaps some of these emotions were projected on the way the interviews were conducted and the data subsequently analysed.

Professionally, AS' perspective of a junior doctor often prevailed over her identity as a psychologist and she approached this research project with a practical focus in mind trying to pinpoint issues that could be changed and improved rather than to purely look for psychological constructs in the data. Theoretical frameworks were used while working with the data and this was to facilitate future practical application of the findings.

Finally, this was AS' first qualitative project. The little experience she had before possibly influenced the way interviews were conducted, particularly in the early stages of the study. Someone with more experience in qualitative research, and specifically in research into sensitive topics might have handled the interviews differently. AS felt she needed some time to gain confidence in her own skills and feel comfortable probing participants about more personal issues.

### Ethics Approval

The ethical approval for the project was sought and received from the East of Scotland Research Ethics Service (REC1) (13/ES/0129) as well as from the School Ethics Committee at the School of Medicine, University of St Andrews (MD10852).

## Results

### Participants

Participant characteristics are presented in Table 1.

**Table 1:** Participants' characteristics.

**Characteristic**	**Number (%)**
**Current Age (Years)**	
≤ 30	1 (4%)
31–35	11 (46%)
36–40	6 (25%)
41–45	6 (25%)
**Cancer Diagnosis**	
Breast	11 (46%)
Cervical	6 (21%)
Ovarian	4 (17%)
Uterine	4 (17%)
**Time Since Diagnosis (Years)**	
0–2	16 (67%)
3–5	8 (33%)
**Partnership Status At Diagnosis**	
Partnered	18 (75%)
Unpartnered	6 (25%)
*Partnership status at interview*	
Partnered	19 (79%)
Unpartnered	5 (21%)
**Education**	
Less than university education	5 (21%)
At least some university education	19 (79%)
**First Language**	
English	23 (96%)
Other	1 (4%)
**Fertility Preservation**	
Yes	8 (33%)
Artificial reproductive technologies	4 (17%)
Gonadotropin-releasing hormone agonist injections	2 (8%)
Trachelectomy	2 (8%)
No	16 (67%)
**Childbearing Status**	
No children	15 (63%)
Child(ren) before diagnosis but not after diagnosis	7 (29%)
No child(ren) before but child(ren) after diagnosis	1 (4%)
Child(ren) before and after diagnosis	1 (4%)

### Themes

We identified five main themes pertaining to treatment-related decision-making experiences and fear of progression and recurrence among young women diagnosed with breast or gynaecological cancer: *Becoming aware of infertility as a potential consequence of cancer treatment; Balancing-prioritising cancer and fertility; Decisions about treatments; Evaluation of treatment decisions;* and *The consequences of treatments*. Subthemes within the themes are described in detail. See Figure S1 for the visual representation of the main themes.

The themes were first organised around the four components of the CSM: “appraisal of health threat,” “illness perceptions,” “strategies to cope with illness,” and “appraisal of coping strategies.”

The appraisal of health threat and illness perceptions are represented throughout the following themes: *Becoming aware of infertility as a potential consequence of cancer treatment;* and *Balancing-prioritising cancer and fertility*. Treatment decision-making processes were conceptualised as a strategy to cope with the illness and are represented in the theme *Decisions about treatments*. The subthemes that were identified within this theme reflect the concepts of the shared decision-making model as it pertains to the clinical settings. Finally, the themes *Evaluation of treatment decisions* and *The consequences of treatments* represent the last component of the CSM—the appraisal of coping strategies.

There were elements of “illness perceptions” in *The consequences of treatments* theme as well. Traditionally, in the CSM, the information gained through the appraisal process feeds back into the coping strategies and allows for their modification as appropriate to a specific situation. In the case of treatment decisions, however, that would be impossible since once treatments had been administered one cannot take back one's decision (e.g., to pursue fertility preservation or not) and opt for a different regimen. One can only cope with the consequences of these decisions made at the time under difficult circumstances. The last theme describes these consequences as well as women's attempts to cope with them in a situation where the change of treatment decisions is impossible.

### Becoming Aware of Infertility as a Potential Consequence of Cancer Treatment

Often women described experiencing a shock upon receiving a cancer diagnosis at a young age. There was a disconnect between the diagnosis and what they perceived themselves to be: a healthy young woman. In addition to transitioning from being healthy to a cancer patient, women also became aware of what this meant for their fertility as a consequence of this disease.

Whether they were diagnosed with gynaecological or breast cancer, most of the women perceived treatments having a potential to be detrimental for their fertility. While for some that was not an issue, others wanted to know what could possibly be done to spare or preserve their fertility. Two scenarios became apparent: fertility discussions were either part of the consultation and initiated by the physicians or they needed to be broached by the women themselves.

For some women, a member of their clinical team brought up the topic of fertility in a consultation. Most women appreciated this, irrespective of whether they were interested in preserving their fertility. They welcomed the opportunity to receive the relevant information and be able to consider what it meant for them. The consequences of those decisions for cancer growth were highlighted by their clinicians.

*I mean the gynaecologist I had was fantastic. Honestly, absolutely fantastic. And he talked through everything. He also said that if I didn't want to go through a hysterectomy just now he could monitor it over a period of time, they could try and give me … oestrogen I think it was and to see if I would … if I could conceive over a period of time and they would help me … But he then told me the consequences of doing that, which is the cancer could grow quicker, you know it might be that I could conceive but I couldn't carry a child, loads of different things. He explained everything fully, gave me the pros and cons and then you know, sent me away to think about it*.

P06, womb cancer diagnosed at 35, no children

When fertility discussions constituted a standard part of a consultation, women had a chance to express their preferences without having to broach the topic themselves. However, that was not always the case. When fertility was not a standard part of the consultation, the onus of initiating the discussion was on women or their relatives.

*So, when I was* first *diagnosed my husband just happened to … say … “Will it affect our chances of having children?” and I'm so glad he thought of asking that ‘cause I … I just wouldn't have asked it. ‘Cause … ‘cause obviously I had lots of other things going on in my head*.

P15, breast cancer diagnosed at 32, no children

On occasion, women had to assume the responsibility for getting informed regarding fertility preservation. They had to be the ones to start the conversation and at times even force their consultants to engage in the discussion about the fertility aspect of their treatment.

*Yeah, it was Dr. [name] who was my* first *oncologist [inaudible], but that was only like I said after I … stood up and said “Is there anything we could do to preserve the fertility?” […] He wasn't willing to discuss it. It was only when I approached it … and then he said he would go away and think about it and look at my case. And it was only when he looked at my case notes and realised I was young and not like 50*—*40 and was only … like a … grade I and grade Ia that he decided to send me for the clinical trial*.

P02, womb cancer diagnosed at 32, no children

In those cases, women often felt that fertility was not important to their physicians and was treated as an “*add-on”* or something extra and not part of standard care. Some even questioned whether fertility would have been discussed at all had they not broached the subject themselves.

### Balancing—Prioritising Cancer and Fertility

Faced with a life-threatening illness, women weighed the importance of fertility against the desire to survive the disease. Through this process they formulated their priorities which were used to guide their treatment decisions.

Women underwent a process of “balancing-prioritising cancer and fertility” in their decisions. Regardless of how important fertility was at the time of diagnosis, most women reported that they wanted to give themselves the best chance at surviving the cancer and prevent any future recurrence.

*Because I was worried maybe if it was gonna be growing to something … more serious. And I was worried it was gonna turn into cancer, so I just wanted everything cleared out*.

P22, borderline ovarian tumour diagnosed at39, no children Some of the participants were prepared to undergo the most aggressive treatments to ensure that no stray cancer cells were left behind even if that meant having to deal with the treatment side-effects and increasing the risk of losing fertility to cancer.

Women who already had children, whether they considered their family complete or not, felt their existing children and getting better for them were their primary concern. Even if fertility preservation was presented as an option, they felt they could not afford an attempt at the potential cost of worsening their prognosis.

*We were very much of the mind, that we have to try our best to … for me to stay alive for my son, for my child and, you know, then not … so that kind of meant that … we said that preserving fertility just wasn't an opt … you know, getting … doing an IVF effectively wasn't an option*.

P10, breast cancer diagnosed at 32, 1 childbefore and 1 child after diagnosis

Women who did not have children were the ones for whom finding this balance between preserving their fertility and the desire to survive cancer was the most difficult since the two were often valued as equally important. However, even this group of participants admitted that it would only make sense to preserve fertility if they were at some point well-enough to actually become pregnant and have a child.

*And he's [the oncologist]… he's always said, which I agree, it's a balance of … it's alright having a baby but you've got to be there, to be around to bring it up (laughter). … It's quite a stark way of saying it*.

P01, breast cancer diagnosed at 32, no children

The balancing-prioritising process certainly differed between women who considered fertility important at the time of their cancer diagnosis and those who did not. However, within the group of women for whom fertility was important, this process also differed between women diagnosed with breast vs. gynaecological cancers. The difference appeared to be related to the distinct consequences that treatment decisions had for the two groups.

For women with breast cancer, receiving chemotherapy did not automatically mean infertility but meant that their post-cancer fertility status would be uncertain. Although chemotherapy could hasten the menopause, if their menstrual cycles returned after chemotherapy, they could still conceive naturally. Therefore, even without fertility preservation, there was hope that their fertility would remain unaffected by cancer treatments. Additionally, in the case of breast cancer, fertility preservation was a process separate from cancer treatments. Women could choose to undergo fertility preservation as an additional procedure which involved a separate decision-making process to the one about cancer treatments. Although potentially not neutral to their cancer prognosis, for women with breast cancer, fertility preservation was not directly related to their cancer treatments.

For women with gynaecological cancers, on the other hand, the two were intrinsically intertwined and therefore the desire to preserve fertility and potentially forgo some of the cancer treatments could have a much bigger impact on their prognosis and survival. There was also no element of uncertainty—once they had radical treatment, their chance of carrying a pregnancy was taken away permanently.

These differences could potentially explain why women diagnosed with gynaecological cancers were much more hesitant while progressing through the balancing-prioritising process. Despite these difficulties however, most of them did choose to go forward with treatments, even at the cost of fertility.

Only one woman positioned herself in opposition to other participants and clearly stated that her priority was her fertility and save it if at all possible, even at the cost of her longer-term prognosis. After weighing the pros and cons she eventually decided to opt in for a radical trachelectomy instead of a hysterectomy.

*The long-term survival wasn't … my longer-term prognosis didn't really ever enter my mind. I would say it did for my family, it did for my partner. I think it may have been there vaguely in the background for me but all I wanted to do was that my fertility wasn't taken away and that my desire to carry a child wasn't taken away. And I didn't want it taken away by cancer if I could at all help it*.

P13, cervical cancer diagnosed at 31,no children.

### Decisions About Treatments

Although women might have prioritised their fertility and survival in different ways, there were similarities in terms of the processes that all of them went through. Also, even though some of the decisions needed to be made at the time of diagnosis and others later, the processes involved in both were very much alike. Henceforth, these main processes are presented for all the participants and all the decisions together under the following subthemes: *Informing vs. involving others;* and *Alignment of treatment preferences between women and their physicians, and its consequences*. Where differences occurred between treatment decisions and decisions relating specifically to fertility preservation or between decisions made at the time of diagnosis and those made later, these are presented within the subthemes. Factors specific to fertility preservation and interrupting the tamoxifen treatment are summarised separately under the subtheme *Specific considerations related to immediate fertility preservation and tamoxifen*.

### Informing vs. Involving Others

Physicians were automatically involved in the decision-making process. Irrespective of the type of treatment women were referring to, the vast majority considered their consultants' opinion to be the most important factor that swayed or even dictated their treatment-related decisions entirely. Yet, there were also other people such as partners and parents whose opinions women took into consideration while making decisions.

There was a clear difference between the degree of involvement of the significant others in the decisions that concerned only cancer treatments and the ones that could also potentially impact on fertility. Treatment decisions, in general, were made between the patient and her clinical team. Women informed their parents and partners about what was going to happen rather than sought their advice. Although family members lent their support to patients' decisions, they rarely played an active role.

*I come from a medical family so of course I spoke to family members and discussed what my consultants were talking to me about […] but I was solely guided by my consultants*.

P14, breast cancer diagnosed at 27, no children

On the other hand, decisions that involved fertility were more often discussed with the significant others. Partnered women often described these decisions as joint decisions. Since fertility was something that couples negotiated between themselves, women considered it important for their partners to partake in decision-making which could potentially affect that aspect of their relationship. Negotiating when to stop the tamoxifen in order to conceive was also described as a joint decision. Partnered women wanted to establish their priorities as a couple and make a decision in line with those priorities, regardless of its final outcome.

*We sort of decided between us that, yes, we did want a family, we wanted that chance. So rather than it being sort of completely taken away from us … at least we'd have the opportunity*.

P15, breast cancer diagnosed at 33, no children

With respect to interrupting the tamoxifen, women specifically emphasised the importance of partner's involvement because of the consequences (e.g., increased risk of cancer recurrence) that such a decision could carry.

*I think he [partner] would have been happy for me to just go and make a decision but I so much felt like I needed that to be a joint decision. If … the shit hits the fan basically … I couldn't ever have him saying “You kind of … you wanted this, you went off and did it” kind of … to me that wasn't … I wasn't comfortable with that … it had to be what we kind of all wanted*.

P10, breast cancer diagnosed at 32, 1 childbefore and 1 child after diagnosis

Women who were in the early stages of their relationships felt that cancer brought forward the discussions and decisions about having children together with their new partners and they did not necessarily feel comfortable with that. Yet, they still did prefer to make decisions about treatments potentially affecting fertility jointly with their partners.

*Not only was it kind of absorbing the fact that I had cancer, it was also putting my partner and I in a position of, well we haven't really spoken about this because we haven't been together a great deal of time … to suddenly, “do we want to have children?” I know I did and we had spoken about it briefly but not to the point of* “*well am I gonna lose my fertility or keep my fertility?.” So, we did have some time to think about it. And it just … to me it was always everything that I wanted to do, to keep fertility. Yeah and we agreed that that would be what we would do*.

P13, cervical cancer diagnosed at31, no children

Single women often consulted with their parents and sought their advice regarding the treatment options.

*I spoke to my dad and he obviously then … said to me … he said “I'd rather you still be with me that having kids. I want you to be healthy.” And he kind of reassured me … and said to me, you know “Have the full operation and you can always adopt, you can always do fostering and stuff like that.”*

P22, borderline ovarian tumour diagnosed at39, no children.

### Alignment of Treatment Preferences Between Women and Their Physicians, and Its Consequences

With respect to fertility, women engaged in the prioritising-balancing process described in the *Balancing-prioritising cancer and fertility* theme. This process enabled women to clarify the value fertility had for them at the time of diagnosis and incorporate it into the decision-making processes about treatments involving the fertility aspect accordingly. Women's preferences, however, were not sufficient to guide treatment decisions. The priority their doctors gave to fertility and patients' childbearing desires equally played a role in the treatment decision-making process. The extent to which the priorities of these two parties involved in the process were in line with each other affected the decision-making.

For women for whom fertility was not an issue, the situation was fairly straightforward since neither they nor their physicians had to factor it into the treatment plan. For women who wished to consider fertility while making treatment decisions and who were under the care of physicians who acknowledged their priorities, the situation was similar and boiled down to discussing the available options and drawing the treatment plan around them.

The situation became more complicated for women who considered fertility while making decisions and who were under the care of the physicians whose priorities differed from theirs. Some women clearly considered preserving their fertility equally important to treating cancer whereas physicians treated it as an “*add-on*.” This created a confusing situation for women who, on the one hand, wanted to follow their consultants' lead and accept the treatments that were suggested to them and on the other, prioritised their fertility differently from their physicians. While many women in this situation ended up accepting treatments suggested by their physicians, some went against the advice they received or consulted another physician.

*So within the* first *week of me being diagnosed I was referred by my oncologist to a gynaecologist at the hospital. And I went to see him and it was, it was quite an awful meeting really because he basically said he wasn't happy doing anything with me because I had oestrogen-positive breast cancer … which, it was just a really awkward meeting, my partner was there with me and we thought it felt like a bit, like we're being interviewed about our relationship and he was very down on it all and said that he would not do anything at all until after I had chemo and I was sort of trying to explain “Well, I've been told that actually … the chemo is going to affect my eggs and my ovaries possibly and my fertility so shouldn't we try and do it before and …” And anyway he just wasn't, he wasn't interested and wasn't going to help me at all. […] A friend of the family went to a consultant [fertility specialist] so my partner and me went to see him, I think this was like the day before my surgery. So it was all like a real mad rush to get it done. And he said “Yeah don't worry at all.” He was brilliant actually. He'd said that he'd treated other women with tumours and there was a pill I could take during the IVF process that would keep my oestrogen levels down and he was just really good and really sympathetic and just sort of gelled with him very quickly*.

P01, breast cancer diagnosed at 32, no children

On the other side of the spectrum were women who opted in for the most aggressive treatments. Some of them saw this as the only way to restore their quality of life which deteriorated because of the symptoms they had prior to diagnosis. However, the most frequent reason for wanting radical treatments was the fear that by doing less, some of the cancer cells would be left behind and the cancer could eventually recur.

*If I hadn't had chemotherapy … there would be a higher risk of recurrence so, I think everything that was thrown at me and everything that was on offer … it can only be positive because you want to throw everything at it*.

P07, breast cancer diagnosed at 39, no children

The physicians acquiesced to patients' preferences regarding treatments as long as they thought these were reasonable. Women's preferences and their consideration were therefore tempered by their physicians' perception of need for treatment.

### Specific Considerations Related to Immediate Fertility Preservation and Tamoxifen

In addition to above influences, there were certain factors women spoke about that related specifically to fertility-related decisions. These included institutional issues, the timing of the initial treatment decisions, and the length of time participants needed to be on the tamoxifen before they could try for a pregnancy.

The availability or services and efficiency of the referral pathways acted as facilitators to receiving fertility sparing or preserving treatment. However, not all women who wanted to take advantage of these services were easily able to do that. Even though assisted conception services for cancer patients are available under the health care scheme, some patients could not get an appointment on time or were disqualified from their use based on age or type of diagnosis. Some of these patients decided to organise a consultation privately. The lack of experience in navigating through the private healthcare system while trying to set up a fertility appointment added to their burden at the time of diagnosis. Cost of the procedures was another issue they had to resolve before pursuing fertility preservation privately.

*So I did feel like in those 3–4 weeks of … from being diagnosed I was going pretty much every day to see an oncologist, or for a blood test or for a different scan or … that every day was taken up with … preparing for my operation and lots of medical appointments … and then on top of that I'm having, I was having to research and try and find somebody to help me [with fertility]. And that was very, very difficult and exhausting I suppose. […] sat trying to get funding, or sat trying to get an appointment for this and that. So, the whole process could have been very much made a lot easier for me and if there was … someone to go to*.

P01, breast cancer diagnosed at 32, no children

Timing of the decisions and haste with which they needed to be made constituted further challenges. Women's impression was that cancer treatment was needed urgently. They explained how their physicians stressed the importance of them getting their treatments as soon as possible and without undue delays. Although time pressure did not necessarily affect the decisions for women with gynaecological cancers who could opt for fertility sparing surgery (e. g., trachelectomy), it was a barrier for women who wanted to take advantage of the assisted conception services.

*When we saw doctor [name], on that* first *time she said, “Look, we can … I can put you forward for egg collection and IVF but that's gonna be another month to 6–8 weeks that I don't particularly want to wait based on your diagnosis. So unless you are absolutely dead set on that, my advice is that we start treatment straight away.”*

P16, breast cancer diagnosed at 32, 1 childbefore cancer

Although in a different way, time also played a role in decisions regarding the tamoxifen. Women's biggest issue when considering whether to stop the tamoxifen was the length of time they should take it for before they could safely interrupt it to conceive. Neither the research, nor the opinions of the doctors were clear with respect to that which made women uncertain as to what the best course of action would be.

*There hasn't been that much that I can find on the Internet, and articles, medical articles about the risks of re-occurrence with coming off tamoxifen before you are advised to and trying for a baby and the effects of hormones on you etc. So, trying to find that information, reading it through and then sort of making that informed decision is important to both of us*.

P01, breast cancer diagnosed at 32, no children.

### Evaluation of Treatment Decisions

The over-riding feeling among women irrespective of the type of decisions they made was that these decisions were right for their particular circumstances. Most women who preserved fertility were grateful they were able to do this. Women found comfort in that their reproductive choices were still theirs as opposed to being entirely out of their hand because of cancer treatments. They also expressed relief at avoiding the regret that they could have potentially felt, had they not acted to preserve fertility. All these women, however, felt well and as far as they were concerned their actions to preserve fertility were not in any way detrimental to their health. Only one woman who had recurrence scares subsequently to her treatment questioned her decision about trying to preserve her fertility at all cost.

*And it was … almost now made me feel like it was the right decision back then in July and August to have the trachelectomy but with complications that have come up and scares that have come up from them, I now feel a bit like a ticking time bomb in that it was right then but I have elements of doubt as to whether perhaps a hysterectomy may have been … a better option?*

P13, cervical cancer diagnosed at 31,no children

Similar to women who preserved fertility, those who did not also felt they made the right decisions regarding their course of treatment. Some found making those decisions easy. Others, despite finding them less straightforward felt that at least they were in control of what was happening to them. One woman, however, felt that she made a mistake by deciding to undergo the treatment whereby her fertility was permanently lost.

*I wish I hadn't done it [had hysterectomy]. It was the biggest mistake in my life*.

P05, womb cancer diagnosed at 31, 1child before cancer.

### The Consequences of Treatments

Irrespective of whether women decided to preserve their fertility throughout cancer treatments, their treatment decisions inevitably had consequences for their post-cancer lives. Fertility-related consequences of cancer treatments are discussed in the subtheme *Cancer-related factors controlling reproductive choices*.

### Cancer-Related Factors Controlling Reproductive Choices

Cancer diagnosis and treatments, irrespective of whether the participant decided to preserve fertility, changed the context of women's reproductive decisions. It took away this spontaneity and brought about additional cancer-related external and internal factors that constrained the realisation of women's fertility-related plans. The external factors included dependence on healthcare professionals and other people to help women either conceive or become a parent through alternative means. Fear of recurrence was an internal factor that acted as a barrier to having children.

Women who pursued artificial reproductive technologies observed that their embryos could only be released to them after a certain amount of time had passed since their treatment.

*I don't think you're allowed to have those embryos released prior to* 2 *years after treatment*.

P07, breast cancer diagnosed at 39, no children

Although they were in a position to make a decision whether to use them, they were not in control of when that would happen. Not only were the healthcare professionals involved in deciding when to release the embryos to the patients but also in carrying out the procedure of the embryo transfer which meant that their assistance was crucial for women.

*I think fertility is the big* one *because it's just taken away the … the sort of … I suppose being able to spontaneously think about having a family. That has to be now more of a … more steps in place before being able to do that*.

P15, breast cancer diagnosed at 33, no children

Since women's decisions regarding the length of time they should be on the tamoxifen were also highly influenced by the advice they were given by their physicians, the time of their eventual pregnancy was again only partially within their control.

*Once we'd started the IVF cycle and I went back to see my surgeon, at the time he said that I'd need chemo, he then said “Well the evidence shows that tamoxifen is more effective for 10 years” so in my mind then I was thinking* “*Well we're [inaudible] eggs collected we have, you know, we're gonna have embryos but I'm not gonna be able to do anything with them because in 10 years” time I'll be, you know, 44.” Originally when we started thinking about doing IVF cycle … and it would have been 5 years on tamoxifen, that kind of would have been fine cause I'd be sort of 39ish so …*

P15, breast cancer diagnosed at 33, no children

Women who underwent trachelectomy for cervical cancer noted multiple possible pregnancy complications that awaited them should they decide to conceive. They were aware that they would require help from the obstetric services to carry the pregnancy and deliver safely. Although these women preserved the ultimate choice of whether to have children, at the same time cancer diagnosis deprived them of the full control over their reproductive decisions. The help of the healthcare professionals became an inherent part of their reproductive choices.

Additionally, women who received the trachelectomy felt as if by the fact that they were offered this procedure, they were also somehow expected to eventually conceive. They were either given a specific timeframe within which they should try for a child or reminded by their physicians that the procedure was done in view of them getting pregnant at some point.

*On more than* one *occasion by more than* one *person it's been suggested that this operation was given to me almost, and in fact* one *professional used that expression … it was given to me … because of the situation I was in, you know, 31 and childless kind of thing. And … they almost, I kind of … I get the impression I'm meant to be grateful for that. I mean, don't get me wrong, I'm grateful for the fact that and the end of the day it saved my life and it was the best option. But it's almost like by not having a child yet I am … I don't know what the best way to put it is. It's almost like I am insulting them by not seeing it through*.

P08, cervical cancer diagnosed at 31,no children

Even women who could not or decided not to preserve fertility noted that their reproductive choices were to a certain extent medicalised. Pursuing surrogacy or adoption depended not only on their wish to do so, but also on their health status and being free of cancer for a specific length of time.

*Like I know I've still got options of like adoption and like a hope that I can still go down this route. I know you've got to be cancer-free for 5 years. And I just hope that I can …*

P02, womb cancer diagnosed at 32, no children

Women who wished to pursue alternative parenting routes also feared the process of their parenting competencies being assessed by other people—a situation that would not have occurred had they not had cancer. For them the ability to extend their families was limited by other people's judgement—potential surrogate mothers in the case of surrogacy, or social services in the case of adoption.

As opposed to these external factors, fear of recurrence was an internal factor which also affected women's plans to have children. Whether they were thinking about biological or alternative parenting, women questioned if it was responsible to have a child knowing that cancer could come back at any time. Some of them thought it would be selfish to pursue pregnancy.

The threat of cancer recurrence was of particular importance to women who were diagnosed with breast cancer. They often linked their disease to hormonal issues and therefore perceived interrupting tamoxifen in order to conceive as potentially increasing their risk of recurrence. They stressed the importance of not “*cutting corners”* with their endocrine treatment to avoid a situation whereby driven by a desire to have a child they would provoke a recurrence and eventually leave a child without a mother. Women who already had children before cancer questioned whether they had the right to take the risk extending their families at the potential cost of their existing children's well-being.

*Yeah … well I do worry about … like … is there a risk of it coming back and … and … and … leaving a child without a mother is an awful thought … and whether that's not a responsible thing to do*.

P04, breast cancer diagnosed at 29, no children.

## Discussion

The aim of this qualitative study was to gain an in-depth understanding of young women's cancer treatment decision-making and the extent to which, as well as the reason why, their decisions were influenced by fertility issues and fear of cancer recurrence and progression. The study was guided by the CSM and shared decision-making model therefore the findings are discussed within these theoretical frameworks.

Our data showed that women's responses to the cancer diagnosis often involved a shock and a sense of disbelief of having cancer at their age. The formation of illness perceptions as a result of the cancer diagnosis soon revolved around its consequences, particularly in relation to fertility. Discussion with their physicians on the impact of treatment on fertility and how fertility could be preserved was expressed as important part of their experience.

Women often appreciated having their physicians initiate a conversation around fertility preservation. Although for some women these discussions occurred in the course of their first consultation and were initiated by a member of their clinical team, for others this was not the case and these women were often disappointed by their physicians trying to avoid the topic and treating it like an “*add-on.”* This is in line with the findings from the literature which suggest that providing women with fertility-related information gives them the sense of agency and control over their lives (Snyder and Tate, 2013) while withholding the information from them engenders the feelings of lack of control and powerlessness (Niemasik et al., 2012; Kirkman et al., 2014). In our study, all of the participants received the relevant information. Nonetheless, some of them reported that they had to take the responsibility for initiating the discussions and felt that had they not done that, the topic might have been ignored.

The literature suggests that upon learning about fertility-related consequences of cancer treatments women engage in the process of finding a balance between survival and fertility (Pellegrini et al., 2010; Gorman et al., 2011; Lee et al., 2011; Hershberger et al., 2013b). The findings of this study align with this concept. Regardless of whether fertility was important to the women, engaging in a balancing and prioritising process enabled them to consolidate their perception of illness and clarify their values with respect to the outcome they wanted to achieve through treatment (to preserve fertility or not). This, in accordance with the CSM, allowed them then to devise appropriate coping procedures and action plans to undertake.

Shared decision-making model (Charles et al., 1997) assumes that for the shared decision-making to occur four conditions need to be fulfilled. First, there needs to be at least two participants in the decision-making—the patient and the physician. However, Charles et al. (1999) specify that this is the minimum number and emphasise that other people such as family members can also be involved. Second, both (or all) parties need to be willing to participate in the process in the sense that both (or all) agree to share the decision-making. If one side does not wish to participate, the decision-making cannot be shared. Third, the information needs to be exchanged between the physician and the patient. The information here encompasses not only the medical knowledge and opinions about different treatments on the part of the physician but also patient's opinions and values that he or she wishes to take into account while making treatment decisions. The information exchange usually happens through the deliberation process where all opinions are weighed and reviewed. Finally, through negotiations, the treatment decision needs to be reached and agreed upon by all parties involved in the process. The assumptions of the shared decision-making model do not preclude the patient from simply agreeing to the treatment suggested by one's physician. However, if one feels coerced to do so, then the process of the decision-making cannot be considered shared.

Women in our study sought healthcare professionals' advice and wished to be guided by experts with regard to their treatment choices which is in line with the first two assumptions of the shared decision-making model. While there is evidence suggesting that young women are more likely to want to participate in cancer treatment decision making (Kane et al., 2014), as well as be active players in the decision-making process compared to their older counterparts (Hamelinck et al., 2018), research also underlines the importance of eliciting patient's preference as to their role in the decision-making process (Weber et al., 2013; Hamelinck et al., 2018). The concordance between patient's desired and actual level of involvement in the decision-making process, as opposed to the level of involvement alone, is likely to be associated with the satisfaction with treatment choice (Keating et al., 2002).

This is also echoed in our study. While both the patients' and physicians' preferences played an important role in the decision-making process in this study, it was the concordance between them that proved to be critical. When women's and their physicians' preferences with respect to fertility were congruous, the decision-making took an unproblematic course. However, when women's preferences with respect to fertility preservation differed from their physicians' priorities, accommodating them in the decision-making process seemed to become more problematic. In the latter situation, two scenarios were most common. One involved women following the expert's advice at the cost of their own fertility-related preferences. In the other, women acted in accordance with their priorities, even if that meant searching for second opinions or going against the will of their physicians.

For the majority of women who may have had particular preferences with respect to fertility, the desire to follow the expert's advice overrode their priorities and dictated their treatment decisions. However, these women also exhibited an understanding why physicians were suggesting treatments which, while lifesaving, could affect fertility. They accepted that fertility was a price they needed to pay to survive their diagnosis. Their physicians took the time to explain that to them. While there might not have been the concordance between the patients' and the physicians' preferences with respect to fertility in those instances, there was congruence between the patients' expectations regarding treatment-decisions and the physicians' practise styles. In their review Kiesler and Auerbach (2006) suggest that it is the latter that matters in terms of satisfaction with the decision-making processes and subsequent psychological outcomes.

In our study, having a trustworthy relationship with the physician facilitated women's decision-making processes. This type of a relationship was usually achieved through open communication, particularly with respect to fertility issues. While physicians were willing to discuss various treatment options, the initiation of fertility-related discussions was often up to the patient. This mirrors the findings of several other studies that looked at fertility preservation among patients with cancer where women had to bring the topic up themselves suspecting that it would not have been addressed at all otherwise (Yee et al., 2012; Corney and Swinglehurst, 2014; Kirkman et al., 2014).

Some women in our study perceived fertility-related communication as far from ideal. Research has shown that physicians frequently have negative preconceptions about initiating fertility discussions and suggesting fertility preservation in the cancer setting (Goossens et al., 2014). As fertility preservation is a time-sensitive issue among women with breast cancer, and strictly related to cancer treatment among women with gynaecological cancers, clinicians may feel that for some patients pursuing any type of fertility preservation is not a viable option and therefore is omitted in discussions and shared-decision making. There is evidence that clinicians are more likely to involve patients in sheared decision-making where equal treatment options providing an actual choice exist (Kane et al., 2014).

However, omitting fertility-related discussions completely for the fear of disagreement between the physician's and the patient's values and excluding it from the shared decision making may have opposite to the desired effect. Occasionally in our study, when the physician was reluctant to discuss fertility, women for whom it was an important topic changed their healthcare providers, even if that meant eventually having to pay for the services.

Finally, evidence shows that women who are unsure of their fertility preferences at the time of diagnosis are more inclined to follow their physician's advice with respect to fertility preservation (Snyder and Tate, 2013). If this advice is not in favour of fertility preservation, it could potentially lead to situations where some women opt against it even if their particular circumstances allow for it. This emphasises the need for the physicians to create an open-minded and non-judgmental environment for the patients to at the very least be able to discuss their fertility concerns and clarify their desires with respect to post-cancer childbearing. The failure to do so in this study may not have resulted in missed opportunities at preserving fertility, however, led some of the women to change their physician or go against their physician's advice to ensure that their priorities were accounted for.

Healthcare professionals were not the only people women wanted to include in the decision-making. Many of the partnered women wished for their partners to be involved in the decisions which could have impact on fertility. They often described these decisions as “*joint*.” While this makes for a complex triadic relationship, a recent review by Gonçalves et al. (2020) highlights the importance of facilitating partners' involvement in fertility-related decisions in the context of cancer. Improved communication of information between the couple and healthcare providers is suggested to contribute to better decision making and ultimately, mental health outcomes (Gonçalves et al., 2020). In the absence of a partner, some of the single women wished to include their parents in the decision-making processes. Although not specific to fertility, a study by Hubbard et al. (2010) points to other benefits of involving family members in cancer-treatment related decision-making. The findings of this study suggest that family members can act as an additional channel of communication with the physicians as well as aid patients in choosing appropriate treatments.

In this study, we also observed elements of fear of cancer recurrence impacting on treatment decisions. Radical treatments were seen as a way of minimising risk of recurrence. The findings of our study are in line with our review on fear of cancer recurrence among breast cancer survivors showing how these fears can impact decisions about pursuing aggressive treatments as well as post-cancer pregnancy decisions (Ozakinci et al., 2014).

The last phase of the shared decision-making process involves reaching a treatment decision between the physician and the patient. An issue specific to fertility preservation reported by women in this study was the timing of the decisions. Often women only had a very short window to make their decision to avoid delaying their cancer treatment. While this evidence corroborates the findings of the literature that the timing of fertility-related decisions is limited and can act as a barrier (Crawshaw et al., 2009; Kirkman et al., 2013; Snyder and Tate, 2013; Garvelink et al., 2015), it also reveals the preferences that physicians had with respect to their patients' treatments, namely that delaying cancer treatments to preserve fertility was not advisable.

According to the CSM, outcome appraisal is an integral part of the process of adaptation to a health threat. The information gained throughout this process feeds back into the organisation of a health threat perception. The altered representation acts as new baseline to modulate subsequent coping strategies which promote adjustment to the illness. Since this study concentrated specifically on treatment-related decision-making as a strategy to cope with cancer diagnosis, the outcome appraisal pertained to the evaluation of the treatment decisions made by the participants and their physicians.

The majority of women in this study felt that decisions they made with respect to treatments were right for their particular circumstances. Two factors possibly contributed to that—the satisfaction with the process of the treatment decision-making, already described earlier and the satisfaction with the outcome of their decisions.

There were two possible outcomes women could have achieved through making their treatment decisions—fertility preservation or lack of thereof. Women who preserved their fertility were generally happy with this outcome although one participant (P13) later questioned whether that was the right thing to do due to cancer recurrence scares she had. Although most women were satisfied with the decisions they made at the time of diagnosis, going back to life as they knew it before cancer proved to be more difficult than some of them might have expected.

In terms of the CSM, the appraisal of coping procedures serves to refine them and improve the adjustment to illness. In many instances it is possible that when one coping strategy fails or is found unacceptable, one can choose from an array of other strategies. This process however, does not fully apply to the case of cancer treatment decisions that can affect fertility. Women only have one chance at making the “right” decision because its consequences are irreversible. Any adjustments to treatment decision-making as a coping strategy can only be made before any actions are carried out. Once the treatments have been administered, the feedback loop is interrupted and any adjustments to treatment decision-making as a coping strategy become impossible. In the post-treatment phase women were left to deal with the consequences of their treatments, pertaining to both fertility and fears of cancer recurrence.

One of the reasons why women decided to preserve fertility was to preserve their choice, however, this proved to be only partially effective. Whilst after cancer women were still in charge of the ultimate decision of whether to have children at all, they were at the same time constrained in how and when to realise their fertility-related plans. Studies reviewing the outcomes of fertility preservation among cancer patients have demonstrated 13–23% utilisation rates of cryopreserved embryos (Barcroft et al., 2013; Dolmans et al., 2015). It would appear that even following treatment, cancer continue to have an impact on reproductive decisions—an irreversible consequence that can prove challenging to women who preserved fertility.

### Limitations and Strengths

Because of the methodology of the study, it has the drawbacks inherent to qualitative research in that its results cannot be easily generalizable. The study sample consisted mainly of well-educated, White, British women and this is the population that the findings could potentially be extended to. Any extrapolations, particularly to different cultural setting warrant caution.

It is possible that due to the recruitment strategy, especially the online method, participants who were interviewed for this study were a self-selected sample of women particularly interested in the issue of fertility after cancer. This would mean that the findings may apply to other women similarly preoccupied by fertility in the context of cancer.

In this study, we used face to face and online methods to approach and invite women to take part. Tackling the differences between these two groups was not the focus of this study, however, reflecting back on the results the following could be observed:

1. Among participants recruited using the face to face method in the NHS clinics were both women who were and those who were not interested in preserving their fertility at the time of cancer diagnosis. Hence, this group was potentially more representative of the population of young women diagnosed with cancer. Qualitative inquiry does not strive to be generalizable in statistical terms but rather to provide an insight into a particular phenomenon (e.g., in the case of this study it was treatment-related decision-making in the context of fertility), therefore representativeness of the sample can be considered less of an issue in qualitative studies compared to the ones using quantitative approach. However, a sample of participants with diverse points of view can provide a more in-depth account of a particular phenomenon and strengthens the analysis in terms of its credibility through the analysis of negative cases. It was additionally observed that, in this study, women who were recruited via the NHS had a rather positive experience of how their fertility issues at the time of diagnosis were addressed. Although clinicians were not informed which of their patients eventually participated in the study and all data were anonymised, it is possible that women who had negative experience with treatment provision were less inclined to take part fearing that their accounts could be made known to their healthcare providers and this in turn could affect the care they were receiving.
2. As opposed to the participants recruited via the NHS, the majority of those who were recruited online reported some issues with how their fertility concerns were addressed at the time of their diagnosis. This could be related to the fact that being informed about the study outside the context of direct healthcare provision (which is in contrast to the women approached for participation via the NHS) potentially made women more confident about and comfortable sharing negative experiences. It is also possible that women recruited online were generally more interested in the topic of the study and therefore less representative of the population of young women with cancer. As they were not directly approached for participation, it can be purported that they either actively searched for information about this particular type of project (e.g., by accessing the research sections of cancer charities websites where advertisement of the project was frequently placed) or their attention was drawn by the project topic as the advertisement appeared on social media accounts of cancer charities.

In conclusion, women diagnosed with breast or gynaecological cancer at a young age undergo a complex process of balancing the wish to survive cancer diagnosis against their desire to preserve fertility to enable them to pursue their reproductive plans. This is best done when decisions regarding treatments that are life-saving but could potentially impact on fertility are shared between women and their physicians. Involvement of partners is also crucial at the stage where fertility is considered. Open communication and expression of one's preferences and values facilitates the decision-making process. Alignment of desired and actual level of involvement in the decision-making as well as the congruence between the patients' expectations regarding treatment-decisions and the physicians' practise styles contribute to the satisfaction with both the process of the decision-making and its outcome. It is important to remember that once treatments are completed, regardless of whether fertility was preserved or not young women struggle with the limitations to their reproductive choices.

These findings need to be interpreted accounting for the limitations of our study. The differences in participants' experiences of and perspectives on treatment-related decision-making in the context of fertility based on the recruitment method (NHS clinics vs. online) could guide recruitment to future oncofertility studies. For projects aiming to obtain a more diverse participant sample and investigate treatment-related decision-making among young women with cancer from a broader perspective it would be advisable to use the clinic-based strategy. For projects that wish to focus on particular issues related to fertility concerns at the time of diagnosis, and the existing issues in addressing them within the clinical setting, an online recruitment strategy would be preferable.

## Data Availability Statement

The dataset generated and analysed during the current study is not publicly available due to the conditions accepted by the participants upon consenting to taking part in the study. This was also approved by the ethics committees involved in reviewing the study [NHS, East of Scotland Research Ethics Service REC1/ University of St Andrews Teaching and Research Ethics Committee (UTREC)].

## Ethics Statement

The ethical approval for the project was sought and received from the East of Scotland Research Ethics Service (REC1) (13/ES/0129) as well as from the School Ethics Committee at the School of Medicine, University of St Andrews (MD10852). The patients/participants provided their written informed consent to participate in this study.

## Author Contributions

This study was conducted as part of AS' Ph.D. project. AS and GO designed the study and AS did the interviews, analysed the data, and wrote up the findings. GO assisted with interpretation of the data and co-wrote this manuscript with AS. All authors contributed to the article and approved the submitted version.

## Conflict of Interest

The authors declare that the research was conducted in the absence of any commercial or financial relationships that could be construed as a potential conflict of interest.
