# Supplementary material for: “Will It Affect Our Chances of Having Children?” and Feeling “Like a Ticking Bomb” —The Fertility Concerns and Fears of Cancer Progression and Recurrence in Cancer Treatment Decision-Making Among Young Women Diagnosed With Gynaecological or Breast Cancer
Source: Front Psychol. 2021 Jun 2;12:632162. doi: 10.3389/fpsyg.2021.632162 (PMC8206503; doi:10.3389/fpsyg.2021.632162)
Supplement: Supplementary file 1 [file Data_Sheet_1.pdf]

1 **Appendix 1. COREQ guidance for reporting qualitative studies applied to this study.**

**Domain 1: Research team and reflexivity**		Addressed in manuscript: Yes/No
Personal Characteristics		
1. Interviewer/facilitator	Which author/s conducted the interview or focus group?	Yes
2. Credentials	What were the researcher's credentials? E.g. PhD, MD	Yes
3. Occupation	What was their occupation at the time of the study?	Yes
4. Gender	Was the researcher male or female?	Yes
5. Experience and training	What experience or training did the researcher have?	Yes
**Relationship with participants**		
6. Relationship established	Was a relationship established prior to study commencement?	No
7. Participant knowledge of the interviewer	What did the participants know about the researcher? e.g. personal goals, reasons for doing the research	Yes
8. Interviewer characteristics	What characteristics were reported about the interviewer/facilitator? e.g. Bias, assumptions,reasons and interests in the research topic	Yes
**Domain 2: Study design**		
**Theoretical framework**		
9. Methodological orientation and Theory	What methodological orientation was stated to underpin the study? e.g. grounded theory, discourse analysis, ethnography, phenomenology, content analysis	Yes
**Participant selection**		
10. Sampling	How were participants selected? e.g. purposive, convenience, consecutive, snowball	Yes
11. Method of approach	How were participants approached? e.g. face-to-face, telephone, mail, email	Yes
12. Sample size	How many participants were in the study?	Yes
13. Non-participation	How many people refused to participate or dropped out? Reasons?	Yes
**Setting**		
14. Setting of data collection	Where was the data collected? e.g. home, clinic, workplace	Yes
15. Presence of non-participants	Was anyone else present besides the participants and researchers?	Yes
16. Description of sample	What are the important characteristics of the sample? e.g. demographic data, date	Yes
**Data collection**		

17. Interview guide	Were questions, prompts, guides provided by the authors? Was it pilot tested?	Yes
18. Repeat interviews	Were repeat interviews carried out? If yes, how many?	Yes
19. Audio/visual recording	Did the research use audio or visual recording to collect the data?	Yes
20. Field notes	Were field notes made during and/or after the interview or focus group?	Yes
21. Duration	What was the duration of the interviews or focus group?	Yes
22. Data saturation	Was data saturation discussed?	No
23. Transcripts returned	Were transcripts returned to participants for comment and/or correction?	No
**Domain 3: Analysis and findings**		
**Data analysis**		
24. Number of data coders	How many data coders coded the data?	Yes
25. Description of the coding tree	Did authors provide a description of the coding tree?	No
26. Derivation of themes	Were themes identified in advance or derived from the data?	Yes
27. Software	What software, if applicable, was used to manage the data?	Yes
28. Participant checking	Did participants provide feedback on the findings?	No
**Reporting**		
29. Quotations presented	Were participant quotations presented to illustrate the themes / findings? Was each quotation identified? e.g. participant number	Yes
30. Data and findings consistent	Was there consistency between the data presented and the findings?	Yes
31. Clarity of major themes	Were major themes clearly presented in the findings?	Yes
32. Clarity of minor themes	Is there a description of diverse cases or discussion of minor themes?	Yes

2 Appendix 2. Interview schedule

General questions

- Could you start by telling me about how your cancer was diagnosed and the treatment process?
- Tell me about your family and friends, and how cancer has impacted on them.

Decision-making

- You have undergone treatment for you cancer. From the perspective you have now, what were the important factors that influenced your treatment decision?
    - Survival, fertility, present family, future family, doctor’s advice, information?

- How do you feel about this/your treatment now?
- How do you feel about the decision process to undergo this treatment?
- How do you think your family and friends feel about this/your decision?

Perceptions of illness

- From the perspective you have now, tell me how you perceive your disease.
  - Tell me about your symptoms.
  - What do you think might have caused your disease?
  - What do you think the consequences of your disease will be?
  - How long do you feel the disease will last?

Fertility

- Tell me what are your expectations regarding parenthood. How do you feel about them?
- Tell me what your fertility means to you. / Tell me about you concerns regarding your fertility.

Ending question

- Do you have any other insights or comments about what we have just discussed that you think are important for me to know?
