# Supplementary figures and images for: “Will It Affect Our Chances of Having Children?” and Feeling “Like a Ticking Bomb” —The Fertility Concerns and Fears of Cancer Progression and Recurrence in Cancer Treatment Decision-Making Among Young Women Diagnosed With Gynaecological or Breast Cancer
Source: Front Psychol. 2021 Jun 2;12:632162. doi: 10.3389/fpsyg.2021.632162 (PMC8206503; doi:10.3389/fpsyg.2021.632162)

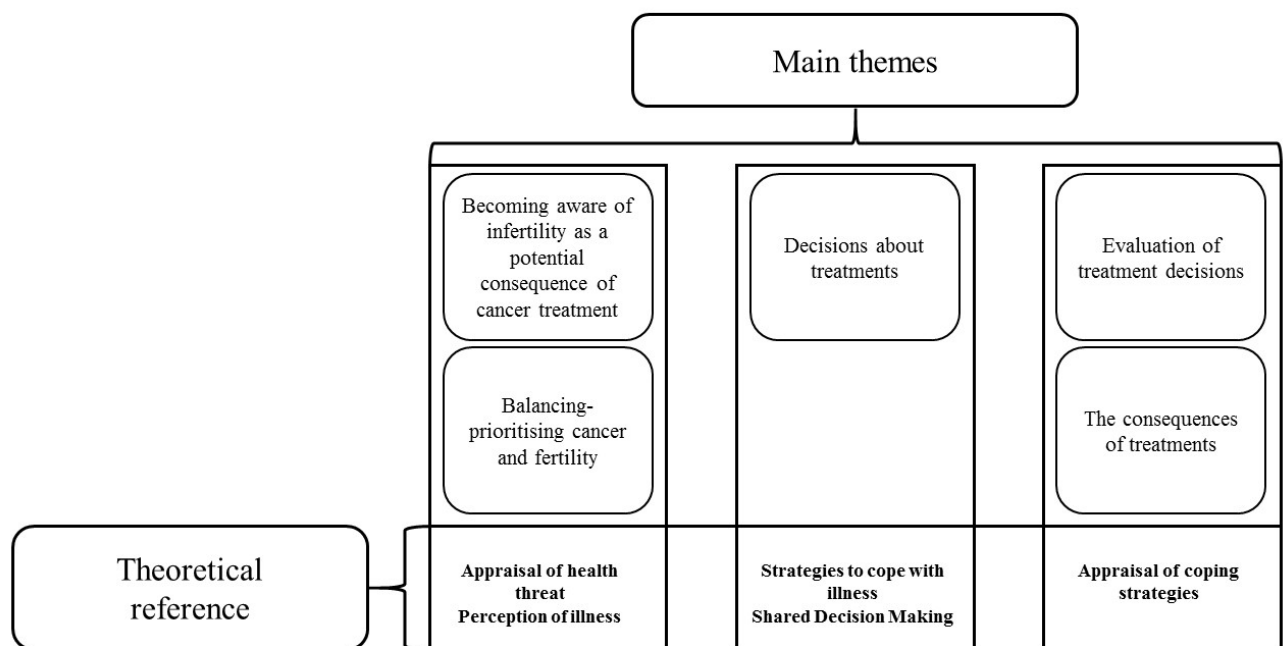

**Figure 1. Visual representation of main themes**

Supplement: Supplementary file 2 [file Image_1.pdf]
